# Biogeography of Speciation of Two Sister Species of Neotropical *Amazona* (Aves, Psittaciformes) Based on Mitochondrial Sequence Data

**DOI:** 10.1371/journal.pone.0108096

**Published:** 2014-09-24

**Authors:** Amanda V. Rocha, Luis O. Rivera, Jaime Martinez, Nêmora P. Prestes, Renato Caparroz

**Affiliations:** 1 Laboratório de Genética e Biodiversidade, Instituto de Ciências Biológicas, Universidade de Brasília, Brasília, Distrito Federal, Brazil; 2 Centro de Investigación y Transferencia de Jujuy, Universidad Nacional Jujuy - CONICET, Jujuy, Argentina; 3 Projeto Charão - Associação Amigos do Meio Ambiente, Carazinho, Rio Grande do Sul, Brazil; 4 Instituto de Ciências, Universidade de Passo Fundo, Passo Fundo, Rio Grande do Sul, Brazil; Bangor University, United Kingdom

## Abstract

Coalescent theory provides powerful models for population genetic inference and is now increasingly important in estimates of divergence times and speciation research. We use molecular data and methods based on coalescent theory to investigate whether genetic evidence supports the hypothesis of *A. pretrei* and *A. tucumana* as separate species and whether genetic data allow us to assess which allopatric model seems to better explain the diversification process in these taxa. We sampled 13 *A. tucumana* from two provinces in northern Argentina and 28 *A. pretrei* from nine localities of Rio Grande do Sul, Brazil. A 491 bp segment of the mitochondrial gene cytochrome c oxidase I was evaluated using the haplotype network and phylogenetic methods. The divergence time and other demographic quantities were estimated using the isolation and migration model based on coalescent theory. The network and phylogenetic reconstructions showed similar results, supporting reciprocal monophyly for these two *taxa*. The divergence time of lineage separation was estimated to be approximately 1.3 million years ago, which corresponds to the lower Pleistocene. Our results enforce the current taxonomic status for these two Amazon species. They also support that *A. pretrei* and *A. tucumana* diverged with little or no gene flow approximately 1.3 million years ago, most likely after the establishment of a small population in the Southern Yungas forest by dispersion of a few founders from the *A. pretrei* ancestral population. This process may have been favored by habitat corridors formed in hot and humid periods of the Quaternary. Considering that these two species are considered threatened, the results were evaluated for their implications for the conservation of these two species.

## Introduction

Speciation remains an important question in science, even after nearly two centuries of research and intense debate owing to the evolutionary theories of Darwin and Wallace [1]. Three models have been proposed to explain the origin of new species of animals with sexual reproduction: allopatric, parapatric and sympatric (for review, see [2]). Allopatric speciation can occur basically in two models: vicariant speciation [3] and dispersion/founder effect [4]. Under the first model, a widely distributed species becomes subdivided into two or more relatively large populations due to extrinsic barriers, and genetic differences begin to accumulate between isolates as each population responds to its own array of selective forces and tracks its ever-changing environment. Under the second model, species evolve through the establishment of small demes often peripherally isolated by dispersing a few founders from an ancestral population.

There are numerous studies of phylogenetic analyses based on molecular data that support vicariance (e.g., [5]), dispersal (e.g., [6], [7]), or both (e.g., [8]) as causes of distribution patterns in Neotropical birds. Similarly, empirical studies have increasingly found dispersion/colonization as a force behind the most common allopatric speciation events [9]–[13]. Estimates of divergence times based on the molecular clock can be used to correlate the time of cladogenesis of species with paleogeographical vicariant events. Depending on the relative ages of species divergence and vicariant events, assessments can be made of whether a dispersal hypothesis (barrier older than species divergence) or vicariant hypothesis (barrier age and species divergence similar) better explains the observed distributions [6]. In many cases, it has been shown that the study group is too young to have been affected by the alleged vicariant barrier, suggesting a recent history of dispersal instead of vicariance of the *taxa* [6], [14]–[16]. In recent years, methods based on coalescent theory have been applied to estimate divergence times among populations and/or species, enabling pure isolation with subsequent independent evolution to be distinguished from isolation with subsequent gene flow [17].

The Spectacled Amazon (*Amazona pretrei*) and the Tucuman Amazon (*A. tucumana*) are Neotropical parrots which were shown to be sister species in phylogenetic reconstructions [18]. However, the Tucuman Amazon was previously regarded as a race of *A. pretrei* [19], but was separated as a full species based on a (most likely erroneous [20]) record of sympatry in the Misiones province of Argentina. These two forms seem to differ in their adult and juvenile plumage, with *A. pretrei* being sexually dimorphic, while the sexes are alike in *A. tucumana* [21], [22]. Different feeding requirements also tend to support the view that these two amazons should be treated as two species, although other evidence is needed to confirm their true relationship [23].

**Figure 1:**
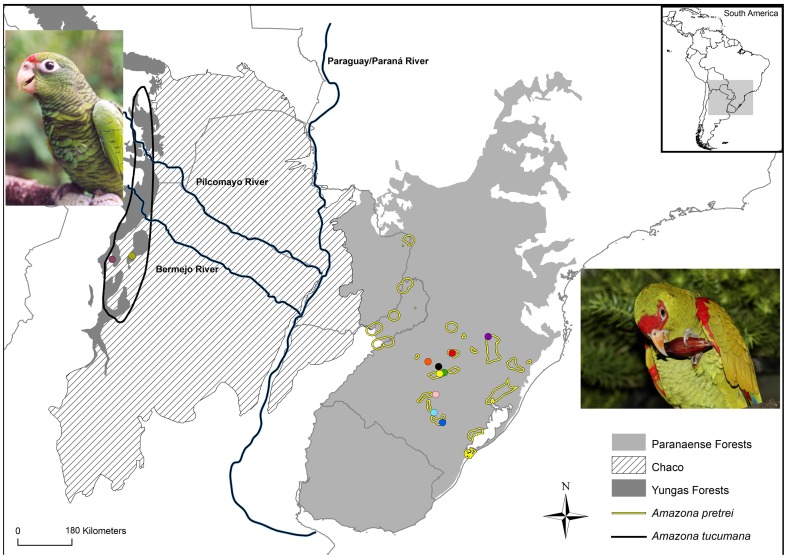
Geographic distribution of *Amazona pretrei* (black circle) and *A. tucumana* (yellow circles). The geographical distribution maps of species were kindly donated by IUCN [71]). Collection localities of samples of *Amazona pretrei* and *A. tucumana* analyzed in this study were indicated by colored dots. For more details of the collection sites, see Table 1.

Currently, the *A. pretrei* distribution area comprises the state of Rio Grande do Sul and southern Santa Catarina in Brazil, with some records in the Misiones province in Argentina (Fig. 1). This species is observed mainly at altitudes between 500 and 1,000 meters in environments marked by the presence of Araucaria (*Araucaria angustifolia*) forests and savannas with Araucaria [24]. *A. tucumana* is restricted to the Southern Yungas forests, a montane forest of the Andes of northwestern Argentina and eastern Bolivia, particularly in areas 1,500–2,200 meters in altitude [25]. These two forest formations are currently separated by approximately 700 km of xerophytic Chaco woodland. The latter is an alluvial plain formed by sediments from the Andes [26] and is part of diagonal open biomes in South America, covering an area of approximately 840,000 km^2^ in northern Argentina, western Paraguay, southeastern Bolivia and the extreme west of Mato Grosso do Sul, Brazil [27]. The flora of this region is considered a relict of the Pliocene or lower Pleistocene, stabilized on salinic soils left after two events of marine transgressions [28], which occurred between 15 and 5 million years ago [29]. Currently, it has a semi-arid climate with intensely hot summers and severe frosts in winter [26]. However, during the interglacial periods of the Quaternary, the Chaco area may have been more humid than today [30]–[32].

According to Nores [33], forests advanced from the southern Yungas and the Paranense region along the Bermejo and Pilcomayo rivers deep enough to form a continuous forest bridge between both regions. Furthermore, Nores [33] noted that some bird species presumably expanded to form a continuous distribution along this continuous forest bridge and became isolated again in the subsequent drier period. This connection-interruption process most likely occurred several times during the Pleistocene and Holocene periods [30], [34]–[36].

Having considered the biogeographical scenario described above and several pairs of species and subspecies with disjunct populations in the Southern Yungas forests and Paraná state (Brazil), Nores [33] described four different levels of bird speciation currently observable in both forests. These different levels of speciation were most likely related to the time elapsed since the species crossed the forest bridge between these two regions. The author classified *A. pretrei* and *A. tucumana* as belonging to an allospecies group, in which differentiation most likely began very early in the wet-dry cycles, although it is possible that isolation and speciation began before the Quaternary period. However, the author noted that future studies applying new methodologies might assist in the review of this classification.

In the present study, we use molecular data and methods based on coalescent theory to investigate whether genetic evidence supports the hypothesis of *A. pretrei* and *A. tucumana* as separate species, and whether genetic data allow us to assess which allopatric model seems to better explain the diversification process of *A. pretrei* and *A. tucumana*. Based on allopatry by vicariance, we should expect that separation of these species occurred before the Quaternary climatic fluctuations, possibly due to the formation of vegetation in the Chaco province estimated to have started during the Pliocene. In allopatry via the dispersion/founder effect or peripheral isolation [4], we should expect that separation occurred subsequently to the formation of the Chaco by a dispersal event of a few individuals which could have been facilitated by the forest corridor, estimated to have occured during the Pleistocene. Finally, considering that the two species in this study are considered to be threatened at a global level [37] or endangered at a national levels [38], [39], the results have been evaluated for their implications for the conservation of these species. For endangered species, knowledge of these historical processes is critical for correct interpretation of current patterns of genetic variation. Furthermore, this information should guide the assignment of conservation priorities and the implementation and elaboration of subsequent conservation strategies.

## Materials and Methods

### Taxon sampling

We sampled 13 *A. tucumana* from two provinces located in northern Argentina and 28 *A. pretrei* from nine localities of Rio Grande do Sul, Brazil (Table 1, Fig. 1). All samples were obtained from nestlings found in natural nests with the permission of the respective environmental agencies (DPMAyRN license n° 082/2006 and APN license n° 04/2008 in Argentina, and SISBIO license n° 25694-3 in Brazil). Only one nestling from each nest was considered for genetic analysis. Three to four drops of blood were taken from each nestling via puncture of the brachial wing vein using needles and glass capillaries, subsequently stored in microtubes containing absolute ethanol. All the samples were stored frozen at the Laboratory of Molecular Genetics and Bird Evolution at the Institute of Biosciences of the University of São Paulo.

**Table 1:** *Taxa* used in this study: species, sample number, sampled locality, haplotype name and GenBank accession number for COI sequences.

Species	Sample	Country	State/Province	Locality	Coordinates	Haplotype	Genbank
*A. pretrei*	3388	Brazil	Rio Grande do Sul	Fortaleza dos Valos	28°48′30.32″S/53°15′34.28″W	pre 7	KF697712
	3390						
	1249			Santana da Boa vista	30°52′58.74″S/53°07′11.97″W	pre 1	KF697709
	1248					pre 6	KF697708
	1234			Jacuizinho	29°01′58.74″S/53°03′04.75″W	pre 3	KF697710
	2257					pre 5	KF697707
	2261						
	1255			Restinga seca	29°50′37.26″S/53°22′15.26″W	pre 5	KF697707
	2257			Carazinho	28°18′13.63″S/52°45′30.35″W	pre 1	KF697709
	2259						
	2260						
	3380						
	3382						
	4699						
	4700						
	4846						
	4701					pre 2	KF697713
	3383					pre 4	KF697711
	3384					pre 5	KF697707
	2267						
	5683						
	5676			Cruz alta	28°37′39.83″S/53°39′00.38″W	pre 5	KF697707
	5693			Caçapava do sul	30°30′40.26″S/53°26′25.26″W	pre 5	KF697707
	2252			Salto do Jacuí	29°04′18.18″S/53°13′21.64″W	pre 1	KF697709
	2253						
	2255						
	4702						
	5675			Barracão	27°41′57.32″S/51°25′32.59″W	pre 1	KF697709
*A. tucumana*	UFG442	Argentina	Salta	Parque Nacional El Rey	24°42′27.56″S/64°37′09.37″W	tuc 1	KF697704
	UFG446						
	UFG440					tuc 2	KF697706
	UFG450					tuc 3	KF697705
	UFG413		Jujuy	Santa Barbara	24°50′06.55″S/65°21′10.19″W	tuc 1	KF697704
	UFG416						
	UFG419						
	UFG420						
	UFG424						
	UFG435						
	UFG430						
	AMCC110751						AY301459
	UFG428					tuc 2	KF697706

### Sample size

For evaluation of whether sequence samples were representative of the genetic constitution of the species, we estimated P = [(k−1)/(k+1)], which represents the probability that a sample of size k and the whole population share the most recent ancestor [40]. P can be interpreted as the probability of the sample being representative of the genetic diversity of the population.

### DNA extraction and sequencing

Total genomic DNA was extracted using a standard proteinase K/SDS and phenol–chloroform protocol [41]. Using a Lambda DNA marker (Biosciences) as a reference, the DNA samples were quantified in a 1.5% agarose gel stained with ethidium bromide.

A segment of the mitochondrial gene cytochrome c oxidase I (COI) was amplified using COIA and COIF primers [42] by polymerase chain reaction (PCR) as follows: final volume of 10 µL containing 0.5 mM of each primer, 0.5 U of Taq DNA polymerase (Phoneutria, CA), 1 mM dNTP, 1× buffer (10 mM Tris-HCl pH 8.3, 50 mM KCl, 1.5 mM MgCl_2_) and 20 to 50 ng DNA. The reactions were subjected to a cycle with a temperature of 95°C for the initial denaturation, followed by 35 cycles at 95°C for denaturation, 53°C for primer annealing and extension at 72°C, ending with one cycle at 72°C for 10 minutes for the final extension. The samples were purified by enzymatic reaction with the enzymes exonuclease I (EXO, USB) and Shrimp Alkaline Phosphatase (SAP, USB). The purified PCR products were sequenced using the DYEnamic ET Terminator kit (Amersham Bioscience) in an automated sequencer ABI 3100 (Applied Biosystems) according to manufacturer's recommendations. The sequences obtained were evaluated for quality and checked for ambiguities using SeqScape v.2.1 (Applied Biosystems). The alignment of the sequences was performed with ClustalW algorithm [43] using the MEGA 5.0 software [44]. The COI haplotype sequences generated and used in this work were deposited in the GenBank (accession numbers KF697704–KF697713).

### Sequence statistics, genetic diversity, and phylogenetic analysis

The nucleotide and haplotype diversity for each species were estimated using the DNAsp 5.10.01 program [45]. The relationship between distinct genetic sequences or haplotypes was evaluated by the haplotype network via median joining method [46] using Network 4.1 (Fluxus Technology Ltd). To verify whether the sequences conformed to neutral expectations of nucleotide substitution, we computed Tajima's D- [47], Fu and Li D* and F* values [48] for each gene using DNAsp 5.10.01.

Phylogenetic relationships among sequences were reconstructed based on the maximum parsimony using MEGA 5.0 [44] and on the Bayesian analyses by Markov Chain Monte Carlo (MCMC) using MrBayes 3.2 [49]. Maximum parsimony analysis was performed considering all the character states as unordered and equally weighted. A heuristic search was implemented with ten random addition sequence replicates, tree bisection-reconnection branch swapping, and a maximum of 100 trees saved per round. To evaluate relative robustness of the clades found in the most parsimonious trees, the bootstrap analysis [50] employed 1,000 replicates using the same heuristic search settings and a maximum of 100 trees were saved per round. Bayesian inference analysis was performed using HKY models. The best-fit model of nucleotide evolution was selected with the Akaike information Criterion (AIC) in JModeltest 2.1.4 [51]. Four Markov chains were run for 2,000,000 generations (sampling every 100 generations) to allow adequate time for convergence. Posterior probabilities of the nodes were computed for all Bayesian analyses across the sampled trees after burn-in. The number of generations required to reach stationary of the posterior distribution was determined by examining marginal probabilities plotted as a time series in Tracer v1.5 [52]. The burn-in period was set to 10%. All MCMCs were run twice to confirm consistent approximation of the posterior parameter distributions. In both analyses, a homologous sequence of *Amazona vinacea* (Genbank AY301462) was used as outgroup. This species was chosen as outgroup based on phylogeny of Russello and Amato [18].

Divergence time and other demographic quantities of the two species were estimated using the isolation and migration model described by Nielsen and Wakeley [17] employing IMa2 [53]. This method uses a Markov Chain Monte Carlo to estimate jointly the posterior distribution of the model parameters and the demographic quantities: θ (N_e_μ, where N_e_ is the effective population size of females and μ is the mutation rate per sequence per generation), M (Nem, where m is the proportion of effective number of migrants per generation) and t (time since population splitting) and TMRCA (time to the most recent common ancestor). For mitochondrial COI, we used the substitution rate of 0.772%/lineage/myr, based on the mean for the Neoaves clade derived in a mitogenomic timescale for birds [54].This rate was converted to 3.8×10^−6^ substitutions/locus/year by multiplying by the number of base pairs of the locus (491), and transforming from millions of years to years. Generation time was set as five years [55]. Several preliminary runs were performed in IMa2 with different priors and heating schemes to find the conditions that allowed proper mixing among chains to avoid local optimum parameter values. When good conditions were achieved, three runs were performed to verify whether the estimated parameters were converging to similar results. Final IMa2 analyses were run for 10,000,000 generations after a burn-in of 10,000 steps using geometric heating, with high heating parameters (g1 = 0.9 and g2 = 0.95), and 20 chains.

Finally, we also evaluated the historical demography by calculating the R2 statistic [56] in DNAsp version 4.0. Significant and low values of R2 suggest demographic expansion if neutrality was not rejected.

## Results

### Genetic variation, haplotype network and phylogenetic analysis

**Figure 2:**
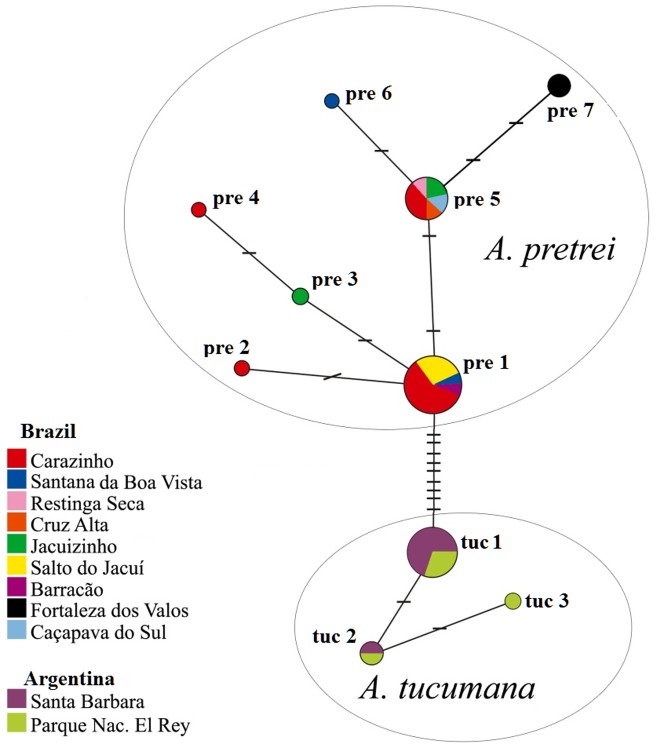
Relationships among the seven *Amazona pretrei* and three *A. tucumana haplotypes* based on the median-joining network of 491 bp of mitochondrial DNA COI sequences. Sizes of circles are proportional to the haplotype frequency. The number of variable sites between haplotypes is indicated by dashes between them.

We analyzed a segment of 491 bp of the COI for all individuals. Translation of the protein-coding gene COI did not reveal stop codons or frame-shift mutations, and third codon positions were more variable than first and second codon positions, as expected in functional genes. In the haplotype network, two haplotype groups were clearly identified, which correspond to the haplotypes of the two taxonomically recognized species (Fig. 2).Ten unique haplotypes defined by seven variable sites (five are parsimony informative) were identified in *A. pretrei*, while only three haplotypes defined by two variable sites (only one parsimony informative) were identified in *A. tucumana*. These two species did not share haplotypes (Fig. 2). Singleton COI haplotypes differed from one another by one to four mutational steps in *A. pretrei*, and by one or two mutational steps in *A. tucumana*. The number of mutational steps separating these species was nine. Considering the genetic variability, *A. pretrei* presented haplotype diversity (D_H_ = 0.68) greater than in *A. tucumana* (D_H_ = 0.41), as well as an average genetic distance (D_M_) three times greater compared to *A. tucumana* (D_M_ = 0.003 in *A. pretrei* and D_M_ = 0.001 in *A. tucumana*). The average genetic distance interspecific was D_M_ = 0.022. When compared to the outgroup (*A. vinacea*), the average genetic distance was D_N_ = 0.057. The COI sequences have an 85% and 93% probability of having correctly sampled the size of the gene genealogy of the *A. tucumana* and *A. pretrei*, respectively. Therefore, our gene samples are considered suitable for the subsequent studies.

**Figure 3:**
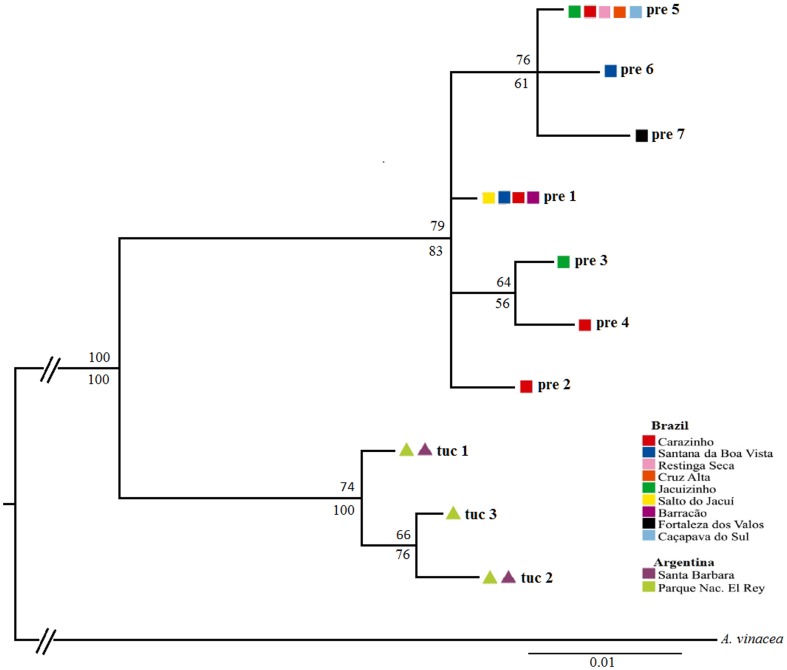
Bayesian tree of 491 bp alignment of mitochondrial DNA COI haplotypes of *Amazona pretrei* (colored squares) and *Amazona tucumana* (colored triangle). Tree is a 50% majority-rule consensus of 18,000 trees. The numbers indicate Bayesian posterior probabilities (above the branches) and maximum parsimony bootstrap (below the branches) values.

The phylogenetic reconstructions based on maximum parsimony (data not shown) and Bayesian analysis (Fig. 3) showed similar results to those obtained by the haplotype network, showing reciprocal monophyly for these two species, with 100% of bootstrap support value and posterior probability of 1.

### Coalescent analyses

Sequence data sets for both species had nonsignificant values (p>0.10) of Tajima's D values and Fu and Li's D* and F* statistics, suggesting that the COI gene is selectively neutral. Therefore, it is appropriate to use it to estimate gene flow and other parameters with neutral coalescent methods.

The effective population size of females for *A. pretrei* and *A. tucumana* was approximately 357,000 and 94,000 individuals, respectively (Table 2). The effective size of the ancestral population could not be estimated properly with the analyzed COI fragment. The divergence between lineages occurred with an absence or low level of gene flow because the effective number of female migrants among populations was estimated to be less than one individual per generation (Table 2). The divergence time of lineage separation was estimated to be approximately 1.3 million years ago (Table 2), which corresponds to the lower Pleistocene. In relation to the time of the most recent common ancestor, it was estimated to be 1.6 million years ago (ranging between 562,016 and 1,996,124 years ago). Assuming a rate of 0.772%/lineage/myr, and based on 5.7% genetic divergence between *A. vinacea* and *A. pretrei*/*A. tucumana* lineages, we estimate that the separation between these lineages occurred at least 3.96 million years ago.

**Table 2:** Demographic and population parameters for *A. pretrei* and *A. tucumana* based on analysis of 491 bp fragment of the mitochondrial gene cytochrome oxidase I (COI) using IMa2 program.

Species	*Ne_F_* (CI)	m1>m2 (CI)	m2>m1 (CI)	*t* (CI)
*A. pretrei*	94,913	0.00		1,339,474
(m1)	(36,192 to 220,785)	(0.00–4.38)		(265,504 to 1,735,876)
*A. tucumana*	27,471		0.00	
(m2)	(2,762 to 135,901)		(0.00–1.86)	

*Ne_F_* - effective number of females; CI - confidence interval of 95%; m1>m2 - effective number of females migrants/generation from m1 to m2; m2>m1 - effective number of females migrants/generation from m2 to m1; *t* - divergence time between *A. pretrei* and *A. tucumana*.

According to the analysis of demographic expansion (R2), the values were not statistically significant (R2 = 0.861, p>0.05), suggesting population stability for both species.

## Discussion

### Species delimitation

Our results indicate that the two amazon species studied here are clearly genetically differentiated. The average genetic distance between these two amazon species was seven fold of those observed within them, including nine fixed nucleotide substitutions. Sequences from the COI fragment indicated a clear subdivision with haplotypes confined to each clade in both network and phylogenetic analysis, indicating reciprocal monophyly for these two species. Coalescent analysis suggested that divergence with little or no gene flow occurred between the two groups in the Lower Pleistocene.

The unified species concept [57] suggests treating independently evolving population lineages as different species if independent lines of evidence are available to support their reciprocal isolation [20]. Thus, the current taxonomic status for these two amazon species is reinforced, since genetic evidence and differences in morphology and ecology (see Introduction) indicate *A. tucumana* lineage is evolving independently from *A. pretrei* lineage.

### Speciation and biogeography

It is not always easy to identify whether two sister species have resulted from vicariance or dispersal events. The vicariance model is supported if the sister species were separated by an intermittent barrier or a barrier that is younger than the estimated age of the ancestral population. In contrast, the dispersion/founder effect model is supported if the sister species were differentiated by means of a permanent barrier, which is considered to be older than the pair of species [58].

According to our analysis, *A. pretrei* and *A. tucumana* diverged with little or no gene flow when the current barrier (xerophytic Chaco vegetation) that separates these two species had already formed (see Introduction). Most likely, this divergence occurred after the establishment of a small population in the Southern Yungas forest through dispersion of a few founders from the *A. pretrei* ancestral population. Thus, this scenario suggests that the allopatry by the dispersion/founder effect model (peripheral isolation) seems to be more likely than by the vicariance model to explain the diversification of these two Amazon species, as discussed below.

According to the phylogeny proposed by Russello and Amato [18], *Amazona vinacea* is the sister species of *A. pretrei*/*A. tucumana* lineage. Considering that *A. vinacea* such as *A. pretrei* occupy the Araucaria forests of southern Brazil [59], it is acceptable to assume that the *A. pretrei*/*A. tucumana* lineage arose in this region. The divergence between *A. vinacea* and *A. pretrei*/*A. tucumana* lineages should have occurred at least 3.96 million years ago when the vegetation of the Chaco was most likely still in training on salty soil, due to the last marine transgression in South America [29], [60]. This non-forest vegetation may have limited the spread of the *A. pretrei*/*A. tucumana* lineage in this period.

Based on our results, the divergence between *A. tucumana* and *A. pretrei* occurred approximately 1.3 million years ago, possibly when the xerophytic vegetation of the Chaco was already similar to current vegetation, limiting the occupation of this area by a typical forest species. It is possible that the formation of more suitable habitat corridors, such as those proposed along the banks of the Bermejo and Pilcomayo rivers across the Chaco during interglacial periods of the Quaternary [33], allowed the passage of amazons through the Chaco arid zone, reaching the wetlands of the tropical forests of the Yungas forest. The isolation between those lineages most likely occurred after the disappearance of these corridors, establishing a condition of isolation between the *A. pretrei* ancestral population and the *A. tucumana* peripheral population. Willis [61] previously also raised the subject of the use of corridors, commenting on the possible use of a south-Andean route (Andean-south route) by *A. pretrei* and *A. tucumana*.

The haplotype diversity observed in *A. tucumana* is lower than in *A. pretrei*, and the relationship pattern among the *A. tucumana* haplotypes in the network, composed by a frequent and internal haplotype and other derivative thereof, can be interpreted as indicative of a founder effect, supporting the peripheral isolation model. However, there is a difference between the sample sizes: the smallest sample has at least 85% genetic variability, moreover, when comparing equivalent numbers of individuals of the two species, variability is still higher in *A. pretrei*. As an example, considering only the 13 spectacled Amazons (same number of *A. tucumana*) from Carazinho, RS, a wider range of haplotypes (D_H_ = 0.64, four haplotypes) was still found in this locality.

Ecological observation also supports the origin of *A. tucumana* in the Pleistocene. *A. pretrei* seems to be dependent on *Podocarpus lambertii* seeds during the breeding season [62] mainly in southern Rio Grande do Sul (savannah with Araucaria), while in the Southern Yungas forests of northwestern Argentina, *A. tucumana* feed their nestlings mainly with *Podocarpus parlatorei* seeds, with 95% of the crop content consisting of seeds (L. O. Rivera, personal observation). Moreover, an increase in nest density was found in years with a high production of *P. parlatorei* fruits. All the populations of *A. tucumana* recorded along its distribution range are found in forests of *P. parlatorei*; this tree is a very good predictor of the presence of *A. tucumana* (L. O. Rivera, personal communication). Ledru et al. [63] found high genetic homogeneity in *P. lambertii*, which occurs mainly in the southernmost latitudes of Brazil, and also noted that such high genetic homogeneity most likely reflects the recent expansion of this genus in the area. This observation, and the affinity between southeastern *Podocarpus* populations and *P. parlatorei* from Argentina and Bolivia, indicates an ancient connection with other South American regions. They suggested that this finding supported the hypothesis of a wider pre-Quaternary distribution of *P. lambertii* on the South American continent, most likely before the formation of the Andes. Thus, it could be possible that a western portion of the continuous extension of *P. lambertii* forest became isolated before the Quaternary and that this forest was then colonized in the Quaternary by the dispersion of some *A. pretrei* individuals, giving origin to *A. tucumana*.

In conclusion, our genetic data indicate that the *taxa* studied here should be considered both distinct and worthy of conservation efforts. However, the scenario of speciation described in this paper was constructed based on a single marker (mitochondrial DNA) with maternal inheritance. Several deviations related to the estimated divergence time and levels of gene flow between populations during this process may be different if other independent markers are observed (see review in Nichols [64]). Thus, it is important to assess this scenario based on other markers to corroborate our biogeographical hypothesis of speciation for these two *taxa*.

### Conservation implications

The conservation of genetic diversity within populations is always desirable to ensure that they are genetically sustainable, particularly in response to future environmental changes. Furthermore, genetic diversity is broadly correlated with population size, hence conservation efforts should seek to maintain or create large populations. The main factors affecting the two amazon species studied here are natural habitat destruction and fragmentation, as well as the illegal trafficking of nestlings and adults [24], [25]. Although these two species are suffering similar human pressures, *A. pretrei* seems to have a better conservation *status* than its sister species.

Systematic monitoring, which began in 1991, indicates that the *A. pretrei* population is stable at approximately 19,000 amazons [65]. Additionally, the genetic variability observed in this species (D_H_ = 0.68) is similar to the one observed (D_H_ = 0.75) in the non-endangered amazon *Amazona aestiva* [55]. This can also be interpreted by comparative analysis made from the analysis of microsatellite loci [65], where *A. pretrei* also shows similar genetic variability to other non-endangered parrots. Thus, the high genetic variability observed in these species can be seen as indicative that anthropogenic interferences have not yet drastically affected its genetic variability. Based on this, it is possible to suggest that the conservation measures to be adopted for this species should focus on the conservation of the remaining forest and restoration of degraded areas, thus ensuring conditions to maintain the current population size and hence genetic variability. In this direction, several actions were proposed in the National Plan of Action for the Conservation of Atlantic Parrots [66] to ensure the conservation of this species, among which we highlight the need for the conservation of remaining forests for nesting and feeding by amazons.

In contrast, *A. tucumana* has a smaller population size in relation to its sister species, with population estimates have identified approximately 8,000 individuals, 80% of which are in Argentina in the northern and central distribution [25], [67]. The low genetic variability observed in this species (D_H_ = 0.41) when compared with its sister species may be mainly the result of its evolutionary history, due to the founding event in the early formation of this species. Furthermore, perhaps in recent centuries, it could have also been influenced by the different negative impacts of human actions, making the species more genetically vulnerable than its sister species does. Thus, conservation efforts for the Tucuman Amazon should prioritize the northern and central regions of Southern Yungas in Argentina, where 80% of the remaining Tucuman Amazon population occurs, with high priority given to the creation of public and private reserves [25]. Furthermore, this species may require actions aimed at increasing the population size, such as eliminating nest poaching and retaining nest and food trees in forestry operations [68], [69]. Finally, the low genetic variability and genetic distinctiveness in *A. tucumana* revealed in our study can be seen as very valuable information given the recent change of the categorization of this species on the IUCN Red List from Near Threatened to Vulnerable [70].
